# The role of autophagy in Graves disease: knowns and unknowns

**DOI:** 10.3389/fcell.2024.1480950

**Published:** 2025-01-06

**Authors:** Hayder M. Al-kuraishy, Ghassan M. Sulaiman, Hamdoon A. Mohammed, Mohammed H. Abu-Alghayth, Salim Albukhaty, Majid S. Jabir, Ali K. Albuhadily, Ali I. Al-Gareeb, Daniel J. Klionsky, Mosleh M. Abomughaid

**Affiliations:** ^1^ Department of Clinical Pharmacology and Medicine, College of Medicine, Mustansiriyah University, Baghdad, Iraq; ^2^ Division of Biotechnology, Department of Applied Sciences, University of Technology, Baghdad, Iraq; ^3^ Department of Medicinal Chemistry and Pharmacognosy, College of Pharmacy, Qassim University, Qassim, Saudi Arabia; ^4^ Department of Medical Laboratory Sciences, College of Applied Medical Sciences, University of Bisha, Bisha, Saudi Arabia; ^5^ Department of Laboratory Techniques, Al-Manara College for Medical Sciences, Maysan, Iraq; ^6^ Department of Clinical Pharmacology and Medicine, Jabir Ibn Hayyan Medical University, Najaf, Iraq; ^7^ Life Sciences Institute, University of Michigan, Ann Arbor, MI, United States

**Keywords:** autoantibodies, autophagy, Graves disease, pathogenesis, thyroid-associated ophthalmopathy

## Abstract

Graves disease (GD), an autoimmune disease affects the thyroid gland, results in hyperthyroidisms and goiter. The main cause of GD is not clearly defined; however, stimulating autoantibodies for thyroid stimulating hormone receptor (TSHR) known as thyroid-stimulating immunoglobulins (TSIs) are the primary proposed mechanism. The TSI activation of TSHRs of thyroid gland results in excessive release of thyroid hormones with the subsequent development of hyperthyroidism and goiter. The cellular process of macroautophagy/autophagy is implicated in the pathogenesis of GD and other thyroid diseases. Autophagy plays a critical role in many thyroid diseases and in different stages of the same disease through modulation of immunity and the inflammatory response. In addition, autophagy is also implicated in the pathogenesis of thyroid-associated ophthalmopathy (TAO). However, the exact role of autophagy in GD is not well explained. Therefore, this review discusses how autophagy is intricately involved in the pathogenesis of GD regarding its protective and harmful effects.

## Introduction

Graves disease (GD) is an autoimmune disease that affects the thyroid gland resulting in hyperthyroidisms and goiter (enlargement of the thyroid gland); therefore, GD is also known as a toxic diffuse goiter (Davies et al., 2020). The name of GD is derived from the Irish doctor Robert James Graves who described a case of GD in 1835. Subsequently, in 1840, the German physician Karl Adolph von Basedow independently described the same features of GD (Weetman, 2003), thus leading to the alternate name of Basedow disease (Leporati et al., 2015) among others. The basic clinical feature of GD includes the symptoms of hyperthyroidism such as weight loss, sweating, tachycardia, and diarrhea (Leporati et al., 2015). Approximately 25%–30% of patients with GD have extra-thyroidal manifestations such as exophthalmos (eye bulging) or Graves opthalmopathy; accordingly, GD is also known as exophthalmic goiter (Cao et al., 2022). The pathognomonic features of GD are hyperthyroidism, goiter, exophthalmos, and pretibial myxedema that not present in other types of hyperthyroidism (Manni et al., 2020). The thyroid-associated opthalmopathy (TAO) develops in GD due to stimulation of thyroid stimulating hormone receptor (TSHR) of fibroblasts and hypertrophy of muscles around the eyes (Neag and Smith, 2022).

The incidence of GD is about 7.5 times more common in women than men. It is more frequent in the age of 40–60 years, although it can happen at any age (Hussain et al., 2017). GD is regarded as the most common cause of hyperthyroidism in the United States (Bartalena, 2013). In addition, GD is often associated with other autoimmune diseases such as rheumatoid arthritis and type 1 diabetes suggesting the immune disorders of GD (Ferrari et al., 2019; Al-Kuraishy et al., 2024b). Of note, genetic causes are also involved in the pathogenesis of GD (Brix et al., 2001). For example, HLA-DRB3/DR3 (major histocompatibility complex, class II, DR beta 3) increases the susceptibility for the induction of GD (Inaba et al., 2016). As well, single gene defects are also linked with the induction of GD. For example, *PTPN22* (protein tyrosine phosphatase non-receptor type 22) and *CTLA4* (cytotoxic T-lymphocyte associated protein 4) gene mutations are implicated in the pathogenesis of GD (Pujol-Borrell et al., 2015). Moreover, the autoimmunity of GD is triggered by viral and bacterial infections due to antigenic mimicry. For example, *Yersinia enterocolitica* has a structural similarity with human TSH receptor and infection by this organism results in the induction of GD (Hargreaves et al., 2013). Likewise; Epstein-Barr virus is considered as a potential trigger of GD (Pyzik et al., 2019).

Although the main cause of GD is not clearly defined, the primary proposed mechanism involves autoantibodies that activate TSHRs; hence, these autoantibodies are known as TSIs (thyroid-stimulating immunoglobulins) (Mathew et al., 2021). TSIs activate TSHRs of the thyroid gland resulting in excessive release of thyroid hormones with subsequent development of hyperthyroidism and goiter (Mathew et al., 2021).

**FIGURE 1:**
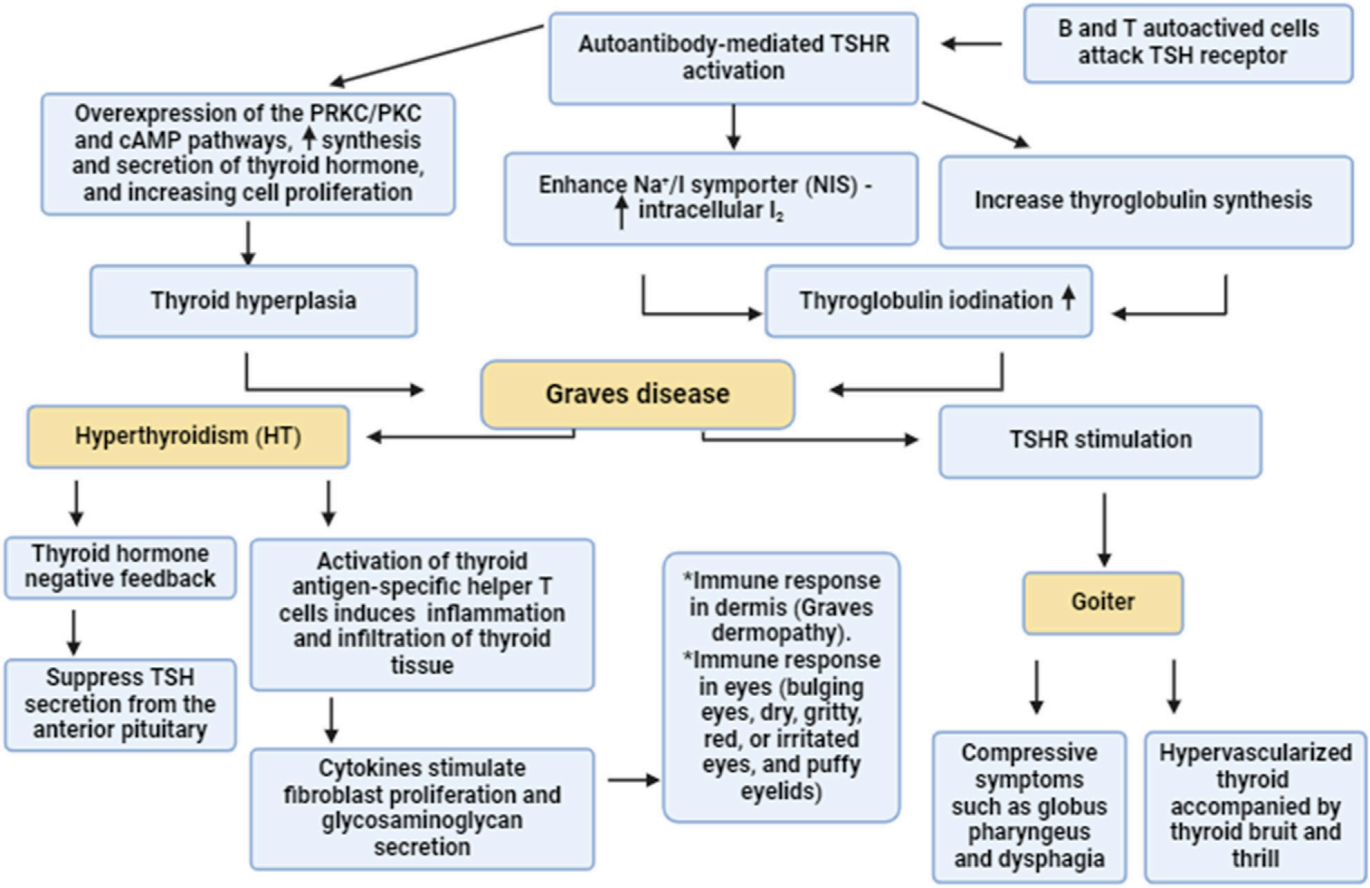
Pathogenesis of GD. Autoreactive B and T cells induce the formation of autoantibodies which activate thyroid stimulating hormone receptors (TSHR). Activated TSHR activates the synthesis and release of thyroid hormones via PKC/cAMP which promote thyroid hyperplasia. In addition, activated TSHR stimulates thyroglobuline synthesis and enhances intracellular accumulation of I_2_ through activation of Na^+^/I symporter. These changes trigger the development of GD which causes goiter and hyperthyroidism.

The underlying mechanisms for the development of autoimmunity in GD are related to the autoactivation of T and B cells with subsequent generation of autoantibodies against TSHRs (Liu et al., 2018). Of note, both cellular and humoral immunity are intricately involved in the pathogenesis of GD. Type 1 T helper (Th1) and Th2 cells are highly involved in the induction of abnormal immune response and the pathogenesis of GD (Antonelli et al., 2020). Th1 through activation of cytotoxic lymphocytes and macrophages affect the proliferation of thyroid follicular cells (Pierman et al., 2021). However, Th2 triggers the production and activation of B lymphocytes and plasma cells resulting in the generation of TSIs against TSHRs (He et al., 2020). In GD, the stimulatory activity of TSI is mainly present in the IgG1 subclass, which is chiefly activated by Th1 cells (Li et al., 2021d). As well, Th1 promotes the generation of TSIs from B lymphocyte via an IL10-dependent pathway (Pierman et al., 2021). Moreover, exaggerated Th17 also activates abnormal TSI in GD (Torimoto et al., 2022). However, regulatory T/T_reg_ cells, which downregulate the abnormal immune response are highly reduced leading to the induction of an abnormal immune response in GD (Fang et al., 2021). In addition, in GD the B lymphocytes are autoactivated due to the downregulation of regulatory B/B_reg_ lymphocytes, also leading to an abnormal immune response and activating the release of TSIs (Wang et al., 2021). Therefore, an abnormal immune response and the development of autoimmunity are involved in the pathogenesis of GD (Figure 1).

Different studies highlight a potential crosstalk between autophagy and the immune response, and the former plays a critical role in autoimmunity (Wu and Adamopoulos, 2017). Autophagy is intricately involved in the expression of intracellular genes, the initial immune response, and cytokine release (Bhattacharya and Eissa, 2013). Autophagy inhibition ameliorates many autoimmune diseases such systemic lupus erythematosus/SLE and rheumatoid arthritis. Conversely, autophagy inhibition exacerbates Crohn disease and psoriasis (Bhattacharya and Eissa, 2013; Wu and Adamopoulos, 2017). It has been reported that autophagy is implicated in the pathogenesis of GD and other thyroid diseases through the modulation of immunity and the inflammatory response (Duan et al., 2019; Chen et al., 2023). In addition, autophagy is also implicated in the pathogenesis of TAO (Duan et al., 2019; Chen et al., 2023). However, the exact role of autophagy in GD is not well explained. Therefore, this review discusses how autophagy plays an integral role in the pathogenesis of GD regarding its protective and harmful effects.

### Autophagy and molecular signaling

**FIGURE 2:**
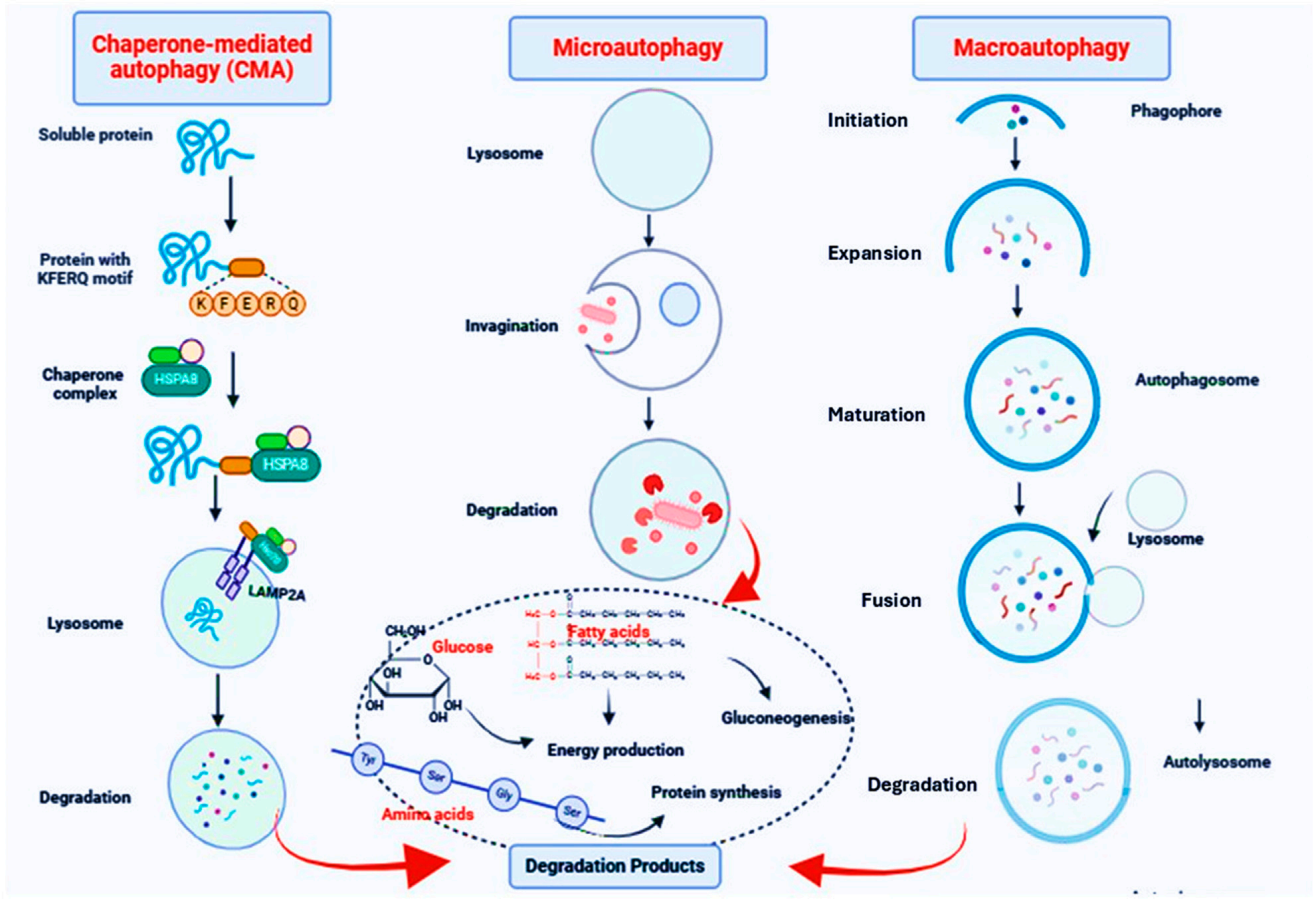
The autophagy pathway. Macroautophagy is initiated by the formation of a phagophore which is sequesters cytoplasmic components and then matures into a double-membrane autophagosome. The autophagosome fuses with an endosome and/or a lysosome to form an autolysosome where the contents are degraded; the resulting macromolecules are then released back into the cytosol. In microautophagy, the limiting membrane protrudes or invaginates at the surface of the lysosome/vacuole by the lateral segregation of lipids and local exclusion of large transmembrane proteins, which is conducted at the small smooth areas with a very low content of transmembrane proteins. In CMA, proteins, the only cargo degraded by this pathway, cross the lysosomal membrane one by one. Not all proteins can undergo degradation via CMA. To be CMA substrates, proteins must contain a specific targeting motif in their amino acid sequence. This motif binds to a cytosolic chaperone (HSPA8), which brings the unfolded substrate protein to the lysosomal surface for internalization and rapid intralysosomal degradation.

Autophagy is an evolutionarily conserved cellular process that promotes the survival of eukaryotic cells in response to different exogenous and endogenous stimuli such as starvation (Ali et al., 2023; Marzoog et al., 2023; Al-Kuraishy et al., 2024c; Sulaiman et al., 2024). Autophagy is involved in the elimination of damaged or superfluous organelles and proteins, recycling the breakdown products that result from their degradation for cellular nutrition (Li et al., 2021b; Al-Kuraishy et al., 2024a). Autophagy plays critical roles in different biological functions in normal and disease states. This process is crucial in the regulation of inflammation, immunity, stress adaptation, cancer, aging and neurodegenerative diseases (Luo et al., 2020). A key feature of autophagy is that degradation occurs through the lysosomal pathway (Cao et al., 2021). There are various types of autophagy that differ in terms of the mechanism as well as substrates (Galluzzi et al., 2017). The predominant form of autophagy, macroautophagy (hereafter autophagy) is initiated by the formation of a phagophore which is sequesters cytoplasmic components and then matures into a double-membrane autophagosome (Broggi et al., 2020). The autophagosome fuses with an endosome and/or a lysosome to form an autolysosome where the contents are degraded; the resulting macromolecules are then released back into the cytosol (Broggi et al., 2020) (Figure 2).

**FIGURE 3:**
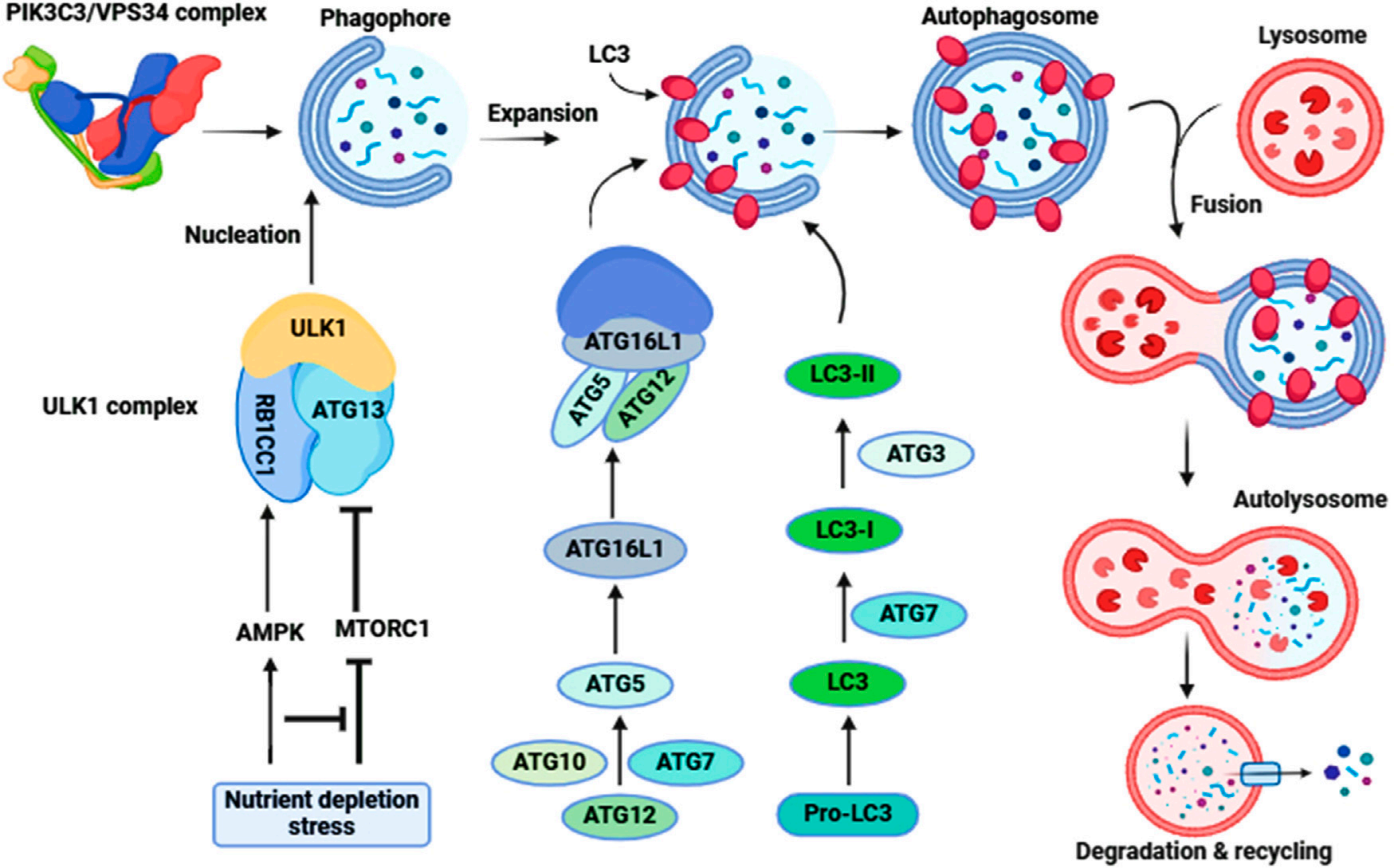
Molecular signaling of autophagy. The ULK1-ATG13-RB1CC1-ATG101 complex is activated to induce autophagy (Initiation). Following the induction of autophagy, an omegasome is formed from the ER by association with ZFYVE1/DFCP1. Next is the formation of a phagophore, which expands to engulf cytoplasmic components, including mitochondria and endoplasmic reticulum (Expansion). Association with the ATG12–ATG5-ATG16L1 complex forms the phagophore. LC3-II localizes to the phagophore membrane at the latter step of autophagosome formation, while the ATG12–ATG5-ATG16L1 complex dissociates from it. Finally, the phagophore membrane is enclosed to form an autophagosome (Maturation). After autophagosome formation, the lysosome fuses with the autophagosome (autophagosome-lysosome Fusion) to form an autolysosome. Intra-autophagosomal contents are degraded by lysosomal hydrolases (Degradation). After formation of the autolysosome, the lysosomal hydrolases degrade the intra-autophagosomal contents, including LC3-II.

Autophagy is coordinated by ATG (autophagy related) proteins. Phagophore formation is initiated by ULK1 (unc-51 like autophagy activating kinase 1)/ULK2 which forms a complex with ATG13, RB1CC1 and ATG101 (Li et al., 2020). This step also requires the class III phosphatidylinositol 3-kinase complex that includes PIK3C3/VPS34, PIK3R4/VPS15, BECN1, ATG14 and NRBF2 (Pavlinov et al., 2020). In addition, BECN1 interacts with other binding proteins such as UVRAG (UV radiation resistance associated), AMBRA1 (autophagy and beclin 1 regulator 1) and SH3GLB1/BIF-1 (SH3 domain containing GRB2 like, endophilin B1) which form various class III complexes (Chang and Zou, 2020). Two ubiquitin-like conjugation systems involving the ATG12–ATG5-ATG16L1 complex, and MAP1LC3/LC3 (microtubule associated protein 1 light chain 3) along with the ATG2-ATG9 lipid transferase-scramblase complex are essential for the expansion of the phagophore (Iriondo et al., 2023); the conversion of LC3-I to the LC3-II lipidated form reflects autophagosome formation (Ueno and Komatsu, 2020). Fusion of autophagosomes with lysosomes requires different components such as UVRAG, VPS proteins and RAB7 (Lőrincz and Juhász, 2020). Autophagy is highly regulated to maintain optimal levels-either too little or too much can be deleterious to cellular physiology. For example, stress and nutrient depletion activate adenosine monophosphate-activated protein kinase/AMPK and inhibit MTOR (mechanistic target of rapamycin kinase) resulting in the activation of the ULK1 and phosphatidylinositol 3-kinase complexes (He, 2022) (Figure 3).

### Role of autophagy in GD

Of note, thyrocyte basal autophagy is essential for the survival of thyroid follicular cells (Kurashige et al., 2019). Kurashige et al. (2019), found that *atg5* gene knockout mice experience abnormal morphology and function of thyrocytes with progressive apoptosis. As well, an imbalance of autophagy and apoptosis triggers the development of thyroid damage in rats (Li et al., 2021c). A reduction of autophagy and augmentation of apoptosis are observed in patients with hyperthyroidism due to excess iodine intake (Xu et al., 2016), and induction of the development and progression of autoimmune thyroid disease (AITD) in animal models is mediated by inhibition of autophagy (Duan et al., 2019). Di-isononyl phthalate/DINP-induced AITD occurs through inhibition of normal autophagy via an MTOR-AKT-dependent pathway. Supporting this finding, inhibition of the MTOR pathway by rapamycin attenuates the development of AITD (Duan et al., 2019). GD is regarded as one of most common AITDs and its pathogenesis is highly affected by the MTOR-AKT pathway (Li et al., 2015). In a GD mouse model, the MTOR-AKT pathway is exaggerated and correlates with signs of hyperthyroidism. Treatment in this model with the antithyroid medication methimazole reduces activity of the MTOR-AKT pathway and mitigates GD pathology (Li et al., 2015; Al-Kuraishy et al., 2021; AlAnazi et al., 2023). Zhang et al. (2023) observed that rapamycin mitigates TAO in patients with GD by inhibiting cytotoxic T lymphocytes, which have an upregulated MTOR pathway. Importantly, isolated IgG from GD patients induces the chemoattractant activity of cytotoxic T lymphocytes via the MTOR pathway (Zhang et al., 2023).

Of note, stimulatory TSIs induce survival and proliferation of thyrocytes, whereas blocking TSIs leads to the inactivation of thyrocytes. Moreover, neutral TSIs, which induce apoptosis of thyrocytes can activate the MTOR pathway and cause a downstream decrease in the proliferation of thyrocytes (Morshed et al., 2010). These findings suggest that activation of the MTOR pathway in AITDs such as GD may be responsible for the inhibition of thyroid autophagy and the progression in the pathogenesis of GD. Thus, restoration of thyroid autophagy may reduce the pathogenesis of GD. Indeed, findings from preclinical studies illustrate that the addition of TSH or the antioxidant N-acetyl-L-cysteine/NAC to rat thyroid FRTL-5 cells activates autophagy and attenuates apoptosis (Morshed et al., 2022). The evidence for autophagy activation is shown by an increase in the levels of SQSTM1/p62, ULK1, LC3A, LC3B and BECN1 as well as PRKN- and PINK-related proteins (Morshed et al., 2022). Therefore, enhancement of thyrocyte autophagy prevents TSI-induced apoptosis in GD. Conversely, Faustino et al. (Faustino et al., 2018) showed that IFNA/IFN-α (interferon alpha) induces AITD through induction of autophagy and lysosomal-dependent degradation of TG (thyroglobulin) in human thyroid cells. Moreover, defective thyrocyte autophagy induces apoptosis of thyroid follicular cells by activating the generation of reactive oxygen species/ROS in patients with Hashimoto disease (Lu et al., 2018). These findings indicate that thyroid autophagy is dysregulated in AITDs including GD.

**FIGURE 4:**
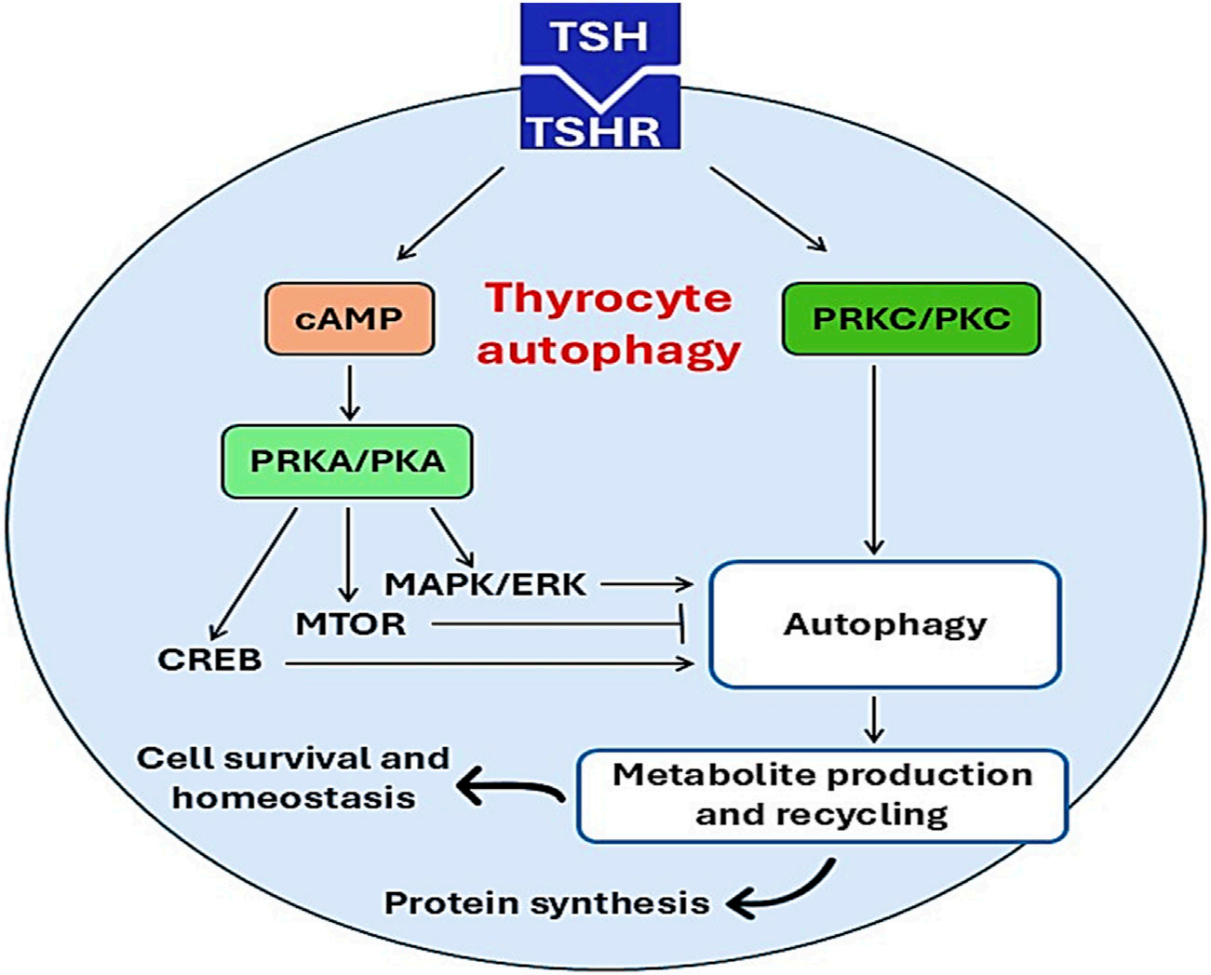
Effects of TSH on thyrocyte autophagy. Activation of TSHR by TSH triggers the activation of thyrocyte autophagy through activation of PKC/cAMP and other signaling pathways. Activated thyrocyte autophagy promotes protein synthesis, cell survival, and nomeostasis.

The physiological level of TSH activates thyrocyte autophagy in PCCL3 cells via cAMP-PRKA/PKA as evidenced by increasing levels of LC3 and SQSTM1; however, thyroid hormones T4 and T3 inhibit thyrocyte autophagy (Kurashige et al., 2020). Moreover, many studies confirmed that TSH activates autophagy in muscles, liver and adipose tissue (Sinha et al., 2015; Lesmana et al., 2016; Yau et al., 2019). However, Xin et al. (2017) illustrated that TSH inhibits autophagy in chondrocytes. Therefore, the effect of TSH on thyrocyte autophagy is difficult to interpret as TSH through activation of PRKA promotes the activation of MAPK/ERK, CREB and MTOR which differentially affect autophagy activation (Figure 4). These findings suggest that the action of TSH and TSI differs downstream as TSI promotes the MTOR pathway whereas TSH mainly activates MAPK/ERK and CREB (Kurashige et al., 2020).

Conversely, GD-induced hyperthyroidism is associated with augmentation of the circulating levels of T3 and T4 which may affect thyrocyte autophagy. *In vivo* and ex-vivo findings demonstrate that thyroid hormones stimulate liver fatty acid β-oxidation through induction of autophagy, and blockade of hepatic autophagy by siRNA which targets *ATG5* inhibits fatty acid β-oxidation (Sinha et al., 2012). Chi et al. (2019) highlighted that thyroid hormones activate hepatic autophagy in different liver diseases such as non-alcoholic fatty liver disease. Therefore, thyroid hormone-induced autophagy can be a compensatory mechanism to control dysregulated autophagy in GD. Remarkably, polymorphism of the autophagy-related gene *IRGM* (immunity related GTPase M) is associated with the risk of GD and other AITDs (Yao et al., 2018) signifying a potential link between GD and autophagy dysfunction. *IRGM* plays a critical role in regulating inflammation by increasing engulfment of apoptotic cells by autophagy (Yao et al., 2018). A case-control study showed that the T allele of rs10065172, A allele of rs4958847, and C allele of rs13361189 are higher in GD patients (Yao et al., 2018). Furthermore, exaggeration of thyroid hormones in GD is linked with the development of oxidative stress, which activates the autophagy flux (Kiffin et al., 2004; Londzin-Olesik et al., 2020). Of interest, oxidative stress is strongly implicated in the pathogenesis of GD (Žarković, 2012). For example, oxidative stress markers are higher in GD patients compared to controls (Ademoğlu et al., 2006). Many studies indicate that oxidative stress through activation of NFKB triggers the development of an autoimmune response in hyperthyroidism (Nandakumar et al., 2008; Makay et al., 2009). NFKB is essential for activation of autophagy (Min et al., 2018) and increases GD risk by 39% (Niyazoglu et al., 2014). Supporting this claim, treatment with the antioxidant selenium reduces the disease severity in GD patients (Marcocci et al., 2011).

These findings proposed that thyrocyte autophagy is dysregulated in GD due to direct effects of TSI and thyroid hormones, and indirectly by oxidative stress and NFKB activation. Moreover, thyrocyte autophagy seems to be inhibited in early GD and activated in late GD to mitigate the inflammatory and oxidative stress disorders.

### Autophagy and GD ophthalmopathy

GD ophthalmopathy (GO) is the most common extrathyroidal manifestation of GD characterized by unilateral (10%) or bilateral (90%) eye proptosis. GO develops due to activation of T cells and TSI directed against retro-orbital tissues, which share antigenic epitopes with thyrocytes (Nabi and Rafiq, 2020). GO as an autoimmune disease leads to inflammation and injury of extraocular muscles and orbital adipose tissues (Bahn, 2010). The occurrence of GO may precede GD in 23% of cases, coexist with GD in 39% and follow GD in 37% (Claytor and Li, 2021). T cell-mediated activation and the Th1 immune response are activated in the early stage of GO, although the Th2 immune response and antibody production are stimulated in the late stage (Chen et al., 2023). These immune responses activate orbital inflammation and differentiation of adipocytes and myofibroblasts (Li et al., 2021a). These immunoinflammatory changes trigger autophagy, which may induce beneficial or detrimental effects according to the disease stage.

The potential role of autophagy in GO had been discussed in different studies; however, the precise role of autophagy in GO was not fully elucidated (Yoon et al., 2015a). In early GO there is marked inflammatory reactions, which induce aberrant autophagy activation (Guo et al., 2020). GO-associated inflammation is linked with autophagy activation as evidenced by increases of ATG5 and BECN1 and higher conversion of LC3-I to LC3-II (Yoon et al., 2015a; Guo et al., 2020). It has been illustrated that autophagy promotes adipogenesis in patients with GO. A case-control study confirmed that ATG5, LC3 and SQSTM1 are increased in orbital fat from GO patients compared to controls (Yoon et al., 2015a) proposing that activated autophagy is implicated in the pathogenesis of GO. Therefore, inhibition of autophagy may attenuate the progression of GO. In fact, it has been established that autophagy inhibitors chloroquine or hydroxycholoroquine attenuate adipogenesis by inhibiting autophagy of orbital fibroblasts (Guo et al., 2020). Similarly, the autophagy inhibitor bafilomycin A_1_ or deletion of *ATG5* inhibits adipogenesis in orbital fibroblasts (Yoon et al., 2015b). Moreover, astragaloside and icariin suppress orbital fibroblasts and adipogenesis through inhibition of autophagy (Li et al., 2017; Li et al., 2018). These findings indicate that autophagy inhibitors are helpful in the management of GO.

Conversely, the MTOR inhibitor rapamycin, which activates autophagy, produces beneficial effects against GO (Roos and Murthy, 2019; Zhang et al., 2023). Rapamycin improves ocular restriction by inhibiting the differentiation of ocular myofibroblasts (Roos and Murthy, 2019). Indeed, the MTOR pathway is upregulated in patients with GO resulting in the induction of inflammation, fibrosis, and adipogenesis. Of interest, low-dose rapamycin mitigates diplopia/double vision in patients with refractory GO by inhibiting CD4-induced inflammation in GO (Zhang et al., 2023). Of note, rapamycin is also effective in different autoimmune disorders such as systemic sclerosis, systemic lupus erythematosus and rheumatoid arthritis (Bruyn et al., 2008; Su et al., 2009). Recently, it has been shown that rapamycin is more effective than steroids in the management of GO (Lanzolla et al., 2022). Therefore, autophagy activators may be effective in the management of GO. These findings highlight the fact that autophagy plays a double-edged sword role in the pathogenesis of GO—it may be protective or harmful according to the different stages of GO.

Taken together, autophagy may be protective against thyroid GD, but it has dual protective and harmful effects. Therefore, additional preclinical and clinical studies are recommended in this regard.

## Conclusion

GD is the most common autoimmune disease of the thyroid gland and is characterized by hyperthyroidism and goiter due to production of TSI. TSI activates TSH receptors of the thyroid gland resulting in excessive release of thyroid hormones with subsequent development of hyperthyroidism and goiter. Of note, autophagy plays a critical role in many thyroid diseases and in different stages of the same disease through modulation of immunity and the inflammatory response. In addition, autophagy is also implicated in the pathogenesis of TAO. Thus this review has focused on how autophagy is involved in the pathogenesis of GD regarding its protective and harmful effects. Thyrocyte autophagy is dysregulated in GD due to direct effects of TSI and thyroid hormones, and indirectly by oxidative stress and NFKB activation. Moreover, thyrocyte autophagy seems to be inhibited in early GD and activated in late GD to mitigate the inflammatory and oxidative stress disorders. Importantly, autophagy plays a double-edged sword role in the pathogenesis of GO, where it may be protective or harmful according to the different stages of the disease. Further preclinical and clinical studies are recommended in this regard.

## Abbreviations

AITD, autoimmune thyroid disease; GD, Graves disease; GO, GD ophthalmopathy; TAO, thyroid-associated opthalmopathy; Th1, type 1 T helper; TSH, thyroid stimulating hormone; TSIs, thyroid-stimulating immunoglobulins.
